# Incidence of mild cognitive impairment in World Trade Center responders: Long-term consequences of re-experiencing the events on 9/11/2001

**DOI:** 10.1016/j.dadm.2019.07.006

**Published:** 2019-09-06

**Authors:** Sean A.P. Clouston, Erica D. Diminich, Roman Kotov, Robert H. Pietrzak, Marcus Richards, Avron Spiro, Yael Deri, Melissa Carr, Xiaohua Yang, Sam Gandy, Mary Sano, Evelyn J. Bromet, Benjamin J. Luft

**Affiliations:** a Department of Family, Population, and Preventive Medicine, Program in Public Health, Renaissance School of Medicine at Stony Brook, Stony Brook, NY, USA; b Department of Psychiatry, Renaissance School of Medicine at Stony Brook, Stony Brook, NY, USA; c United States Department of Veterans Affairs National Center for Posttraumatic Stress Disorder, Clinical Neurosciences Division, VA Connecticut Healthcare System, West Haven, CT, USA; d Department of Psychiatry, Yale University, New Haven, CT, USA; e Unit for Lifelong Health and Ageing, University College London, London, UK; f MAVERIC US Department of Veterans Affairs Boston Healthcare System, Boston, MA, USA; g Department of Epidemiology, Boston University School of Public Health, Boston, MA, USA; h Department of Psychiatry, Boston University School of Medicine, Boston, MA, USA; i Department of Medicine, Renaissance School of Medicine at Stony Brook, Stony Brook, NY, USA; j Department of Medicine, Stony Brook University, Stony Brook, NY, USA; k Department of Neurology, Icahn School of Medicine at Mount Sinai, New York, NY, USA; l Department of Psychiatry, Icahn School of Medicine at Mount Sinai, NYC, New York, NY, USA; m James J Peters VAMC, Bronx NY, USA; n World Trade Center Health and Wellness Program, Department of Medicine, Renaissance School of Medicine at Stony Brook, Stony Brook, NY, USA

**Keywords:** World Trade Center disaster, Posttraumatic stress, Mild cognitive impairment

## Abstract

**Objective:**

This study examined whether World Trade Center (WTC) exposures and chronic posttraumatic stress disorder (PTSD) were associated with incidence of mild cognitive impairment (MCI) in a longitudinal analysis of a prospective cohort study of WTC responders.

**Methods:**

Incidence of MCI was assessed in a clinical sample of WTC responders (*N* = 1800) who were cognitively intact at baseline assessment. Crude incidence rates were calculated and compared to population estimates using standardized incidence ratios. Multivariable analyses used Cox proportional-hazards regression.

**Results:**

Responders were 53.1 years old (SD = 7.9) at baseline. Among eligible cognitively intact responders, 255 (14.2%) developed MCI at follow-up. Incidence of MCI was higher than expected based on expectations from prior published research. Incidence was higher among those with increased PTSD symptom severity, and prolonged exposure was a risk factor in apolipoprotein-ε4 carriers.

**Conclusions:**

PTSD and prolonged WTC exposures were associated with increased incidence of MCI in WTC responders, results that may portend future high rates of dementia in WTC-exposed responders.

The events following the attacks on the World Trade Center (WTC) on September 11, 2001, were cataclysmic. The impacts of these exposures, and their known sequelae, on mental and physical health, remain largely unknown. Many of the more than 60,000 men and women who worked in rescue and recovery efforts at the WTC saw maimed, dead, falling, and dying people and also inhaled dust and smoke containing toxic chemical pollutants during response efforts [1]. Chronic posttraumatic stress disorder (PTSD) remains among the top five conditions reported in WTC-exposed individuals, even two decades after 9/11 [1], [2]. In other populations, PTSD and long-term exposure to airborne pollutants have been linked with increased risk of dementia [3], [4]. Chronic PTSD is a disorder commonly characterized by the stressful re-experiencing of a traumatic event accompanied by a chronically dysregulated stress-response [5], [6] that has been associated with cognitive dysfunction [7], changes in neural responses [8], increased neuropathology [9], reduced hippocampal volume [10], and cortical thinning [11]. Long-term exposure to airborne pollutants has been associated with increased neuropathology [12], [13], dopaminergic changes [14], and incidence of Alzheimer's disease [15].

Research studies of WTC responders have found impairments in cognitive and physical functioning in this population [16], [17], [18], which is concentrated among responders reporting high levels of chronic intrusive re-experiencing stress. In addition, early reports have identified a small association between lengthy exposures to the pile/pit and cognitive dysfunction [17]. To date, prior research has been limited to cross-sectional association studies. This is the first study to longitudinally assess the relationship between PTSD and the development of mild cognitive impairment (MCI). This approach is critical because MCI is heterogeneous, and its implications are most concerning when accompanied by decline in cognitive abilities [19]. The objective of this study was to determine the incidence of MCI in a consecutive sample of cognitively normal WTC responders participating in a clinic-based monitoring study. We hypothesized that incidence of MCI would be more common in WTC responders than in estimates from the general population and that PTSD symptom severity and WTC exposure duration would be associated with increased risk of incident MCI.

## 1 Methods

### 1.1 Setting

All WTC responders are eligible to attend annual monitoring and treatment appointments, for free, by one of the dedicated WTC responder clinics [20]. Each clinic manages cases in their geographically determined catchment area. Stony Brook University (SBU) operates two clinical centers that provide annual monitoring visits to WTC responders residing on Long Island, NY. Prior analyses have found that the SBU clinic monitors responders who are similar in terms of exposures, PTSD burden, and age on 9/11/2001 to the responder population [1]. In 2014, SBU began the first and only prospective study to date to assess indicators of aging in WTC responders by incorporating cognitive assessments into the monitoring visit [18].

### 1.2 Study population

Baseline data collection occurred between 01/01/2014 and 12/31/2015 during regularly scheduled monitoring visits among responders who were fluent in English (Supplementary Fig. 1). The follow-up period was 1.0–2.5 (mean = 1.48) years after baseline assessment with a cutoff date of 07/01/2017. Visit date was recorded for each visit. Eligible responders participated in the initial cognitive assessment (response rate = 95.6%).

### 1.3 Non-WTC comparison population

The recruitment of a non-WTC comparison cohort was outside the scope of this study. Incidence in the general population was determined by completing a random-effects meta-analysis of data retrieved for the present study from two systematic reviews of proximal diagnostic categories that were estimated at 50.10/1000 person-years (95% CI = [38.09–62.11]) among participants with average age of 74.8 years (Supplementary Material).

### 1.4 Ethics

The Stony Brook University Ethics Review Board approved this study (#604113); responders provided written informed consent. All procedures were carried out in accordance with the approved protocols.

### 1.5 Role of the funding source

The funding source played no role in the study design, data collection, analysis, interpretation, or reporting for this study. The corresponding author had full access to these data and final responsibility for the decision to submit for publication.

### 1.6 Measures

#### 1.6.1 Mild cognitive impairment

This study systematically identified MCI in a manner consistent with the *National Institute on Aging–Alzheimer's Association's* definition for MCI [19]. Cognitive status was assessed using the Montreal Cognitive Assessment (MoCA), a test that was designed to identify multidomain MCI [21]. The MoCA has excellent test-retest reliability and is internally consistent [21]. To avoid test-retest biases resulting in increased scores on neuropsychological tests, alternate versions of the test were used at each visit. Because epidemiological analyses relying on the MoCA need to use more conservative cutoffs than those used in a neurological clinic [22], we used a conservative cutoff validated in epidemiological settings (MoCA ≤ 23) [23]. Finally, because memory declines are often reported among individuals with symptoms of anxiety and distress [24], and because one symptom of PTSD is self-reported difficulties with memory, cognitive decline was assessed objectively as decay in cognitive functioning at the follow-up assessment.

Posttraumatic stress disorder symptom severity was assessed at monitoring visits using a WTC-specific form of the PTSD Checklist (PCL-17), which assessed PTSD symptoms related to experiences at the WTC site [25]. To reduce the risk of reverse causation, PTSD symptom severity was measured at enrollment beginning in July 2002. Responders reported the extent to which 17 DSM-IV PTSD symptoms had bothered them in the past month, with responses ranging from not at all (1) to extremely (5). Neurodegenerative conditions often cause neuropsychiatric symptoms in the form of anxiety and elevated depressive symptoms [26]; however, recent work has shown that re-experiencing symptoms are less sensitive to increases in symptomatology seen in cognitively impaired individuals, so the focus in this study was on the symptoms [27]. PTSD symptom severity denotes symptoms summed across the five re-experiencing symptoms in a standard way and scaled to range from no symptoms (0) to maximal symptoms (1) to facilitate effect size comparison.

#### 1.6.2 World Trade Center exposure

To infiltrate the brain, inhaled particulate matter must be vanishingly small (<0.1 μm), round, and smooth [28]. Thus, although most research in this population focuses on pulmonary exposures with large and jagged pieces having the potential to become lodged in the lungs, we propose that inhaled dust that is burned or pulverized over a long period may increase risk of dust filtering into the brain through the blood-brain barrier [17]. No questions in the exposure interview directly address this type of exposure; exposure severity was therefore classified as working on the pile or in the pit where exposures were the most intense and for a prolonged duration (defined as being in the top quintile, ≥15 weeks [29]).

#### 1.6.3 Apolipoprotein genotyping

*APOE*ε4 allele possession is associated with increased risk of dementia, in part because it increases blood-brain barrier permeability [30] and may therefore increase vulnerability to dust-related exposures. DNA was extracted from blood collected during visits at study baseline. SNP genotyping was performed using the Agena iPLEX kit with processing completed at Roswell Park Laboratories. Because of small size (n = 18) of the homozygous-*APOE* ε4/ε4 group, homo/heterozygous *APOE*-ε4 carriers were combined.

#### 1.6.4 Demographics

Age in years was recorded. While sex is not associated with incidence of MCI in the general population [31], sex was also recorded for descriptive purposes. Occupation was dichotomized into nontraditional (e.g., construction workers) versus law enforcement groups. Education was categorized into high school education or less, some college, and university degree. Race/ethnicity was categorized into mutually exclusive categories: white, black, other, or Hispanic.

#### 1.6.5 Physical health

Clinic-recorded diagnoses of all-cause cancer, diabetes, hypertension, and cardiovascular disease were examined. Pre-911 head injury (not due to WTC) was measured using a structured history and categorized as minor (nonconcussive without loss of consciousness), concussive, involving loss of consciousness, or multiple reports across types.

#### 1.6.6 Measures used for exclusion

Diagnoses of the following health conditions made at any time before the second cognitive assessment were retrieved from the responders' clinical records: stroke, Parkinson's disease, Alzheimer's disease, other dementias, and multiple sclerosis. Evidence of a WTC-related head injury was collected using a structured history and by reviewing diagnostic charts identifying a traumatic brain injury occurring at the WTC.

Sensitivity analyses (Supplementary Material) were conducted to clarify modeling assumptions, to examine other potential psychiatric comorbidities and to discuss the relevance of other specifications of PTSD symptoms and diagnosis.

### 1.7 Statistical analysis

Means with standard deviations, and percentages, were used to describe the sample. Bivariate analyses relied on t-tests and nonparametric trend tests to calculate *P* values. Because both PTSD and MCI are common outcomes in this cohort, multivariable analyses in the Supplementary Material and in analyses of attrition reported in the main text relied on log-binomial models to provide unbiased estimates of the relative risk (RR) [32]. Crude incidence rates and age-standardized incidence rates (aIRs; /1000 person-years) were calculated using direct standardization with the U.S. 2000 standard population [33]. Standardized incidence ratios were calculated [34] to compare crude incidence rates to population estimates as noted previously. Cox proportional-hazards regression was used to model incidence [35]; unadjusted associations and multivariable-adjusted associations were reported. Age/sex-adjusted incidence by time figures was estimated using the results from Cox models for different PTSD and exposure groups. All analyses were proportionally weighted to match the responder population [1]. Schoenfeld residuals were used to test the proportional-hazards assumption. Analyses were conducted using Stata IC/14.2 [StataCorp].

## 2 Results

### 2.1 Sample description

After excluding responders with WTC/military-related head injuries or preexisting neurocognitive disorders, the final sample (Table 1) was in their mid-50s, on average, and most participants were male, worked in law enforcement, had more than a high school diploma, and worked six weeks at the WTC site. Applying exclusion criteria resulted in exclusion of 62 responders who had a history of neurocognitive diseases including brain cancer and dementia, and 16 responders with incomplete information. Of those who were eligible, 87.7% completed a follow-up visit within the observational period, 82.8% of whom completed follow-up cognitive assessments. Analyses of the likelihood of attrition revealed that those who were cognitively impaired at baseline were more likely to lack a follow-up cognitive assessment (RR = 1.24, 95% CI = [1.08–1.42], *P* = .002) and also revealed that among those who were cognitively normal, that having a MoCA < 26 was also associated with loss to follow-up (RR = 1.17, 95% CI = [1.02–1.34], *P* = .028). At the initial assessment, 390 (17.8%) responders with MoCA scores indicative of cognitive impairment were excluded from this analysis, while 255 (14.2% of the sample) developed MCI at follow-up. Bivariate analyses identified greater PTSD symptom severity, severe WTC exposure, and presence of *APOE*-ε4 coupled with high exposure as risk factors for MCI. Analyses also identified some non-WTC risk factors for MCI including older age, occupation, and educational attainment.

**Table 1:** Sample characteristics at baseline for the eligible sample and stratified by incident mild cognitive impairment status

Responder characteristics	Total (N = 1800)	Cognitively intact∗ (N = 1545)	Mild cognitive impairment at follow-up (N = 255)	*P*
	Mean (SD)	Mean (SD)	Mean (SD)	
Age, years	53.15 (7.89)	52.91 (7.68)	54.59 (8.92)	.001
PTSD symptom severity	0.13 (0.18)	0.13 (0.17)	0.17 (0.23)	<.001
High WTC exposure, %	10.06	9.39	14.12	.038
High WTC exposure and *APOE*-ε4 carriers, %	2.22	1.49	6.67	<.001
*APOE*-ε4 carrier versus noncarrier, %	21.28	20.84	23.92	.258
Female versus male, %	8.50	8.35	9.41	.591
Race/ethnicity, %				
White	65.96	67.51	56.48	
Black	3.94	3.37	7.45	<.001
Other	22.94	22.39	26.27	.036
Hispanic	7.17	6.73	9.80	.017
Nontraditional responder	42.61	41.29	50.59	.006
Educational attainment, %				
High school or less	49.00	48.54	51.76	
Some college	29.89	29.32	33.33	.838
University degree	21.11	22.14	14.90	.046
Hypertension	29.39	28.74	33.33	.134
Diabetes	10.11	10.36	8.63	.405
Cardioarterial disease	4.50	4.40	5.10	.612
All-cause cancer	16.39	15.86	19.61	.128
Head injury, %				
None	62.67	62.72	62.35	
Minor	8.33	8.28	8.63	.844
Concussion	4.33	4.53	3.14	.356
Loss of consciousness	10.11	10.10	10.20	.938
Multiple	14.56	14.37	15.69	.617

Abbreviations: PTSD, posttraumatic stress disorder; WTC, World Trade Center; SD, standard deviation.

NOTE. Race/ethnicity categories are mutually exclusive; the other race category includes other races and non-Hispanic multirace categories. *P* values derived from two-tailed t-tests derived from bivariate log-binomial regression.

∗ Responders who were cognitively intact at baseline and remained that way at follow-up.

### 2.2 Incidence of MCI

**Fig. 1:**
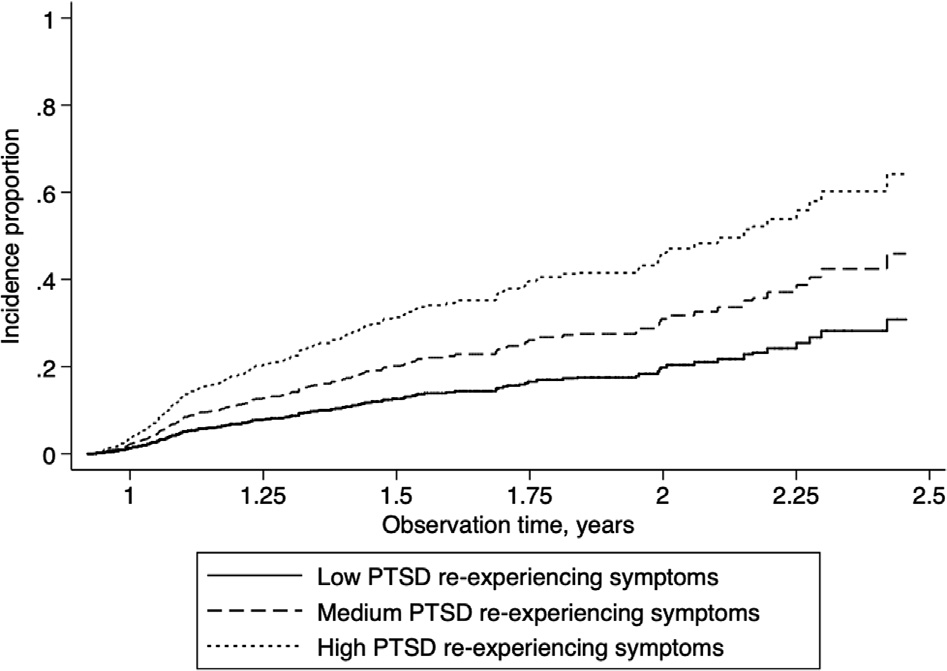
Incidence proportion of mild cognitive impairment over time among responders, separated by PTSD symptom severity groups. PTSD symptom severity was categorized into low (0–0.249; 78.0% of the sample), medium (0.25–0.49; 15.5% of the sample), and high (0.50–1.00; 6.4% of the sample) in this figure. Abbreviation: PTSD, posttraumatic stress disorder.

**Fig. 2:**
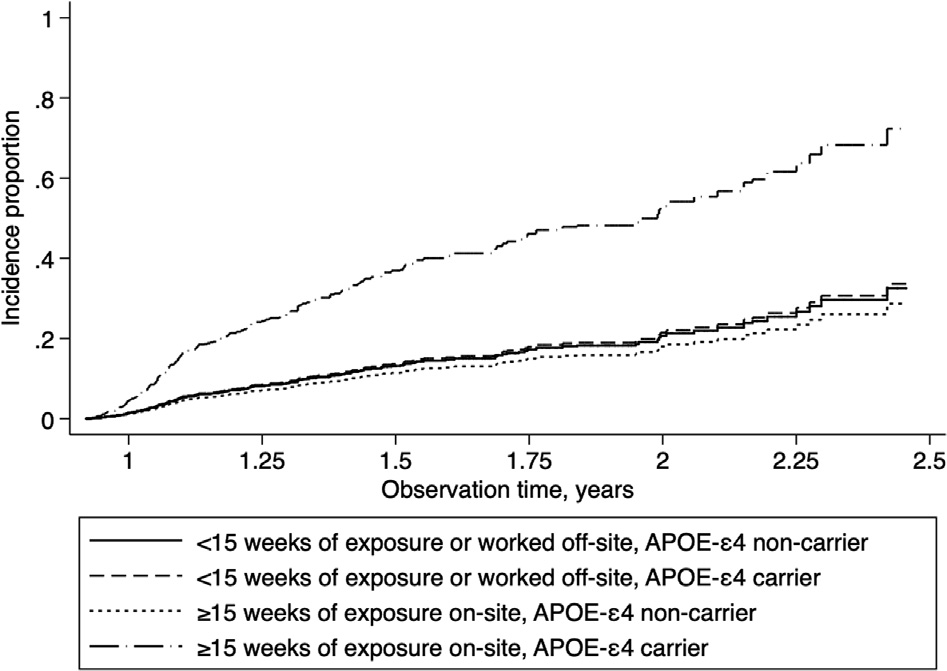
Cumulative incidence proportion showing incidence of mild cognitive impairment among responders, separated by WTC exposure severity and by *APOE*-ε4 allele possession status group. Abbreviation: WTC, World Trade Center.

Crude incidence rates were 108.09/1000 person-years (95% CI = [95.94–122.24]). Age-standardized incidence of MCI was estimated to be aIR = 159.51 (95% CI = [154.81–164.21]) in the WTC responder population. Comparing crude rates to published results (aIR = 50.10/1000 person-years), incidence was higher in WTC responders than in the general population (standardized incidence ratio = 2.16, 95% CI = [1.92–2.41]). Cumulative incidence of MCI was higher among those with higher PTSD symptom severity (Fig. 1), and among responders possessing the *APOE*-ε4 reporting severe WTC exposures (Fig. 2), supporting trends identified in Table 1.

**Table 2:** Unadjusted and multivariable-adjusted hazard ratios examining cumulative incidence of mild cognitive impairment among those who were cognitively intact at first assessment

Variable	Unadjusted		Multivariable-adjusted analyses	
	aHR (95% CI)	*P*	aHR (95% CI)	*P*
Age, years	1.024 (1.006–1.042)	.009	1.021 (1.000–1.041)	.046
PTSD symptom severity	2.974 (1.489–5.94)	.002	2.672 (1.331–5.367)	.006
WTC exposure severity	1.300 (0.889–1.902)	.176	0.925 (0.551–1.552)	.767
WTC exposure severity among *APOE*-ε4 carriers	3.215 (1.965–5.261)	<.001	3.889 (1.788–8.462)	.001
Any *APOE*-ε4 versus no *APOE*-ε4	1.293 (0.957–1.749)	.095	1.043 (0.736–1.479)	.813
Female versus male	1.087 (0.681–1.733)	.727	1.024 (0.637–1.647)	.921
Race/ethnicity				
White	1.000		1.000	
Black	1.584 (0.935–2.683)	.088	1.582 (0.932–2.685)	.089
Other	0.638 (0.47–0.867)	.004	0.718 (0.496–1.038)	.078
Hispanic	1.141 (0.707–1.841)	.590	1.255 (0.769–2.047)	.364
Nontraditional responder vs. law enforcement	0.808 (0.622–1.049)	.110	0.898 (0.656–1.229)	.502
Educational attainment				
High school or less	1.000		1.000	
Some college	1.142 (0.87–1.499)	.338	1.100 (0.799–1.514)	.559
University degree	0.602 (0.421–0.862)	.006	0.686 (0.465–1.013)	.058
Hypertension	1.354 (1.028–1.783)	.031	1.273 (0.948–1.711)	.109
Diabetes	0.972 (0.608–1.553)	.906	0.732 (0.444–1.204)	.219
Cardiovascular disease	1.711 (0.981–2.984)	.058	1.882 (1.058–3.347)	.031
All-cause cancer∗	1.185 (0.842–1.666)	.331	1.042 (0.721–1.504)	.828
Head injury				
None	1.000		1.000	
Nonconcussive	1.196 (0.747–1.916)	.455	1.210 (0.752–1.947)	.433
Concussion	0.809 (0.388–1.688)	.573	0.705 (0.339–1.468)	.351
Loss of consciousness	0.928 (0.606–1.421)	.731	0.810 (0.522–1.258)	.348
Multiple	1.132 (0.787–1.627)	.505	1.089 (0.747–1.588)	.657

Abbreviations: WTC, World Trade Center; PTSD, posttraumatic stress disorder; HR, hazard ratio; aHR, adjusted hazard ratio; 95% CI, confidence interval.

∗ History of all-cause cancer excludes brain cancer. Reference categories labeled 1.000. Multivariable analysis adjusted only for variables shown.

Examining age-adjusted incidence rates revealed similar overall trends: compared to those with low PTSD symptom severity (aIR = 99.77/1000 person-years, 95% CI = [95.47–104.06]), incidence rates increased in responders with medium (aIR = 178.29/1000 person-years, 95% CI = [175.26–181.32]) and high PTSD symptom severity (aIR = 219.05/1000 person-years, 95% CI = [215.97–222.13]). Compared to those with either low WTC exposure severity or who do not carry the *APOE*-ε4 allele (aIR = 114.60/1000 person-years, 95% CI = [110.10–119.11]), those who were severely exposed had higher incidence (aIR = 150.61/1000 person-years, 95% CI = [147.55–153.68]) while those who carried the *APOE*-ε4 allele and had high WTC exposure severity had very high incidence of MCI (aIR = 527.06/1000 person-years, 95% CI = [524.41–529.70]).

### 2.3 Multivariate analyses

Prior results were confirmed first in unadjusted and then in multivariable-adjusted analyses. The proportional-hazards assumption was upheld in these analyses (χ^2^ = 14.8, *P* = .613). Analyses revealed that both higher PTSD symptom severity and *APOE*-ε4 allele possession among severely exposed WTC responders were associated with increased risk of incident MCI (Table 2).

### 2.4 Sensitivity analyses

Additional analyses examined the extent to which predictors including PTSD symptoms and WTC exposures may be explained by inclusion of possible alternative specifications for mental health conditions including diagnosed anxiety and depressive disorders. Consistent with the literature, diagnoses with these disorders were highly associated with increased PTSD symptom severity at enrollment (depression: RR = 13.98 [10.77–18.14]; anxiety: RR = 9.52 [726–12.49]) raising concerns about multicollinearity. Supplemental analyses (Supplementary Material) revealed that the appropriate specification was PTSD symptom severity as measured here.

## 3 Discussion

Individuals involved in the WTC disaster are now at midlife, a time when aging is starting to become clinically apparent. This is the only prospective longitudinal study of cognitive aging in responders who participated in the rescue and recovery efforts following the attacks of 9/11/2001 on the WTC. We found higher than expected incidence of MCI over an 18-month follow-up period when comparing incidence statistics to estimates among unexposed individuals from the general population of individuals who were, on average, 20 years older than those studied here. In addition, this study reported a strong association between prolonged WTC exposure and incident MCI among responders carrying the *APOE*-ε4 allele and further noted that WTC exposures were strongly associated with incidence of MCI among those possessing the *APOE*-ε4 allele.

This study was conducted in a single clinical center that monitors responders living in Long Island, NY. These responders are similar in levels of exposure and PTSD symptom severity to the >60,000 WTC responders (including firefighters, police, construction, media, medical, and volunteer responders) monitored across all the WTC monitoring programs [20]. These findings therefore imply a potentially high burden of MCI in the broader WTC responder population.

The prognosis for MCI in this cohort is unknown. Some cases may develop dementia, whereas others will remain stable and many will resolve. Yet, while the clinical diagnosis of dementia was outside the scope of this study, analyses examining diagnostic accuracy in the *Alzheimer's Disease Neuroimaging Initiative* suggest that 41.9% of older individuals with MCI (MoCA ≤ 23) also have functional limitations sufficient for dementia diagnosis [36]. While not at all definitive, the high level of functional limitations accompanying MCI is concerning because only 1/1000 people aged 45–64 years are expected to have dementia at this age group [37], whereas 41.9% of those with incident MCI in this study would reflect a baseline prevalence of dementia in the range of 74.5/1,000. Studies are now underway to assess the level of functional limitations in this population.

Chronically re-experiencing traumatic memories are foundational symptoms in PTSD and may be crucial when studying the long-term results of traumatic events because these emotionally intense memories link experiences to long-term dysregulation in neurobiological processes [38]. These recalled stressful experiences are fairly common, with more than one-quarter of WTC responders experiencing these symptoms more than a decade after the event [2]. Neuroimaging studies have reported that trauma survivors with chronic PTSD have more rapid hippocampal volume loss [10], reduced cortical thickness [11], increased *β*-amyloid deposition [9], and changes to plasma *β*-amyloid burden [39].

While PTSD was a key risk factor in these analyses, some of the excess risk in this population may be directly attributable to WTC exposures. Compositional studies have identified known neurotoxins in dust collected at the WTC [40]. Building on a prior study, which showed that prolonged work on the pile was associated with reduced cognitive performance [17], we also found a weak association between prolonged exposure that was magnified among responders carrying the high-risk *APOE*-ε4 allele. These results support the potential for dust exposures to infiltrate the brain when blood-brain-barrier permeability is increased as has been reported for *APOE*-ε4 carriers [30] but may also be common in old age [41]. Future analyses should seek to replicate these analyses.

### 3.1 Limitations

Though being the first study of its kind among WTC responders, this study was limited in a number of ways. First, we relied upon a meta-analysis of other incidence studies to provide a comparison population. The best comparison group would be one made up of similarly educated and employed individuals who did not respond. The extreme nature of the WTC exposure meant that most NYPD, who make up 66% of our population, aided in response efforts. Those who did not may be unique in a number of ways, including that they were disabled or otherwise unfit during the response efforts. Nevertheless, while the comparison statistics presented are not optimal, there is a known need to clarify our expectations about the level of incidence expected for these purposes. In addition, we relied upon a short but well-validated clinical examination to identify MCI. This examination could fit the short time window that was available but is not a comprehensive neuropsychological examination and does not allow for domain-specific analysis. Furthermore, despite the fact that we defined MCI and accompanying cognitive decline in a way that is consistent with researchers in the field of Alzheimer's disease, in the absence of neural biomarkers, we cannot ascertain pathogenesis or possible prognosis of MCI in this cohort. Individuals in this study were cognitively assessed for the first time more than 10 years after WTC exposures. Although individuals who were already cognitively impaired at baseline were excluded from this study, the follow-up of cognitive status in this population relying on alternative measures of risk of Alzheimer's and related diseases will be crucial for future studies. There was no time allotted for cognitive assessments to validate cognitive status across all recorded cases. Because of strong symptomatologic overlap between psychiatric disorders, we were not powered to discriminate between mental health disorders in multivariable analyses. In addition, while individual risk for MCI appears to be increasing in this population, there are no comparable data with which to directly compare incidence in such a young population. While this study was interested in MCI [19], a prodrome for Alzheimer's disease, recognizing the youth of the population and the lack of quantitative neuropathology were refrained from applying diagnostic labels in favor of more descriptive language. A final limitation is the reliance on self-reported symptoms from enrollment visits rather than diagnostic information for PTSD. While not always preferable, this focus on symptom domains is in line with the increased interest in symptoms-focused definitions of mental health diagnoses and recognizes the emerging consensus that specific PTSD symptoms may have different long-term consequences and specifically that chronically re-experiencing a stressful event may carry undue influence on neurobiological risk [38].

## 4 Conclusions

This study is the first of its kind in a sample of WTC responders and provides increasing support for the view that WTC exposures may have neurological implications. The long-term risks on health after WTC exposures are largely unknown. In this cohort, the incidence of MCI was more than three times as common as has been previously shown in similar studies. Clinicians and policy-makers need to be aware of the increased risk for early MCI in this population and the utility of monitoring cognitive functioning in the long term.

**Research in Context**

1. Systematic review: We reviewed the literature in PubMed and Google Scholar and found no prior work detailing incidence of mild cognitive impairment (MCI) among World Trade Center (WTC) responders. However, prior studies identified high levels of chronic posttraumatic stress disorder (PTSD), a risk factor for MCI in veterans and Holocaust survivors.
2. Interpretation: In this study of responders without comorbid head injury, crude incidence of MCI was higher in this population than in other prospective studies of individuals aged, on average, 20 years older. WTC-related PTSD, identified at program enrollment occurring as early as July 2002, was a main risk factor for MCI. In addition, prolonged exposure to the WTC site was a risk factor among apolipoprotein-ε4 carriers.
3. Future directions: PTSD is a risk factor for MCI in WTC responders at midlife. Future work should seek to determine the prognosis for MCI at midlife in this cohort and to detail the neuropathological correlates for the disease.
