# Supplementary material for: Incidence of mild cognitive impairment in World Trade Center responders: Long-term consequences of re-experiencing the events on 9/11/2001
Source: Alzheimers Dement (Amst). 2019 Sep 6;11:628–36. doi: 10.1016/j.dadm.2019.07.006 (PMC6733774; doi:10.1016/j.dadm.2019.07.006)
Supplement: Supplementary Material [file mmc1.docx]

**Supplemental Appendix A.**

*Comparison rates*

Establishing a good incidence rate was challenging given the reliance here on a more recent test of cognitive impairment (MoCA) and the lack of comparable studies in this age range. As such, we have here provided the estimated incidence of amnestic MCI in older populations. Studies reporting an incidence rate ^1-13^ were retrieved from a systematic review of sex differences in incidence of MCI ^14^ and from a systematic review on incidence and prevalence ^15^. The mean age across included studies was 74.82 years (SD = 5.39). Additional studies were identified by a reference search of the included studies. The MoCA assesses whether individuals fail across a range of neurocognitive tests, including but not limited to memory tests. As such, we utilized incidence of multi-domain all-type MCI where feasible and cognitive impairment not dementia (CIND) if only that was available. To get incidence of all-type MCI in the whole study, we returned to the manuscripts cited in the systematic review. In all cases, the full-text of these papers was retrieved and the rates were clarified; when only subtypes were reported, all-type was inferred as the summation of all subtypes. Mean age was also recorded, in cases where the paper did not report the mean age, mean age at baseline was retrieved from other papers describing the same population. Only studies reporting incidence rates calculated using person-years, and not percent with MCI at follow-up, were utilized. In cases where incidence rates were reported, but confidence intervals were not, a confidence interval was calculated using standard methods ^16^. Meta-analysis was completed using random-effects estimation because of the potential for significant variability around the estimated mean ^17^, which was substantial among included studies (*I^2^* = 96.8%, P<0.001, and *τ^2^* = 454.71).

**Figure A1.** Meta-analytic results examining overall incidence rates across population studies reporting incidence rates

*Occupational differences:*

Prior work has noted that the risk of PTSD is somewhat lower in law enforcement compared to other responders in this cohort ^18^. As such, we examined the utility of examining the support for the view that the association between PTSD re-experiencing symptoms and CI incidence differed by occupational status by incorporating an interaction between the two. Survival analyses showed no support for the view that the association between re-experiencing symptoms and CI incidence differed by occupational status (*P*=0.875).

*Symptomatology*: The diagnosis of PTSD requires both severe symptomatology and the reporting of functional limitations in addition to the intrusion of stressful memories often characterized as re-experiencing symptomatology, the focus of this and many other studies. We here below examined symptoms of effortful avoidance (e.g., actively avoiding reminders), emotional numbing (e.g., emotionally distancing from life), and hyperarousal (e.g., being ever-aware and on edge). The following table shows unadjusted associations between each of the four PTSD symptom subdomains measured at enrollment in the monitoring program, and then again at the time of diagnosis of CI incidence. Additionally, prior work in this study, and in other studies, has noted that some of these symptoms are very similar to neuropsychiatric symptoms commonly reported as comorbidities and/or indicators of latent neurodegenerative disease. Results showed (Table A1) that the core re-experiencing symptoms for PTSD have a consistent association with CI incidence while the level of association, and significance of the association, increased among those with incident CI justifying the focus on re-experiencing symptomatology in this study.

**Table A1.** Unadjusted associations between posttraumatic stress disorder symptom subdomains and incidence of cognitive impairment

|  |  | PTSD symptoms measured at enrollment visits for the parent monitoring program | | |  | | PTSD symptoms measured at first cognitive assessment | | | |
| --- | --- | --- | --- | --- | --- | --- | --- | --- | --- | --- |
| **PTSD symptomatology** | | **HR** | **95% C.I.** | **P** | |  | | **HR** | **95% C.I.** | **P** |
| Re-experiencing symptoms | | 2.974 | (1.489-5.940) | 0.002 | |  | | 3.499 | (1.884-6.498) | <0.001 |
| Avoidance symptoms | | 1.023 | (0.598-1.750) | 0.933 | |  | | 1.620 | (1.004-2.614) | 0.048 |
| Numbing symptoms | | 1.373 | (0.712-2.647) | 0.344 | |  | | 1.838 | (0.968-3.489) | 0.063 |
| Hyperarousal symptoms | | 1.308 | (0.748-2.286) | 0.346 | |  | | 1.897 | (1.092-3.297) | 0.023 |

Note: HR: Hazard Ratio; 95% C.I.: 95% confidence interval;

*Symptoms versus diagnosis*: We examined clinical (chart) diagnoses of WTC-PTSD in addition to symptom inventories used in this study. In these data, Table A2 found that re-experiencing symptomatology was able to explain the weak (non-significant) association between PTSD diagnosis and incidence of CI.

**Table A2.** Adjusted associations between posttraumatic stress disorder re-experiencing symptoms as well as posttraumatic stress disorder clinical diagnoses and incidence of cognitive impairment

| **PTSD characterization** | **aHR** | **95% C.I.** | **P** |
| --- | --- | --- | --- |
| PTSD clinical diagnosis | 1.355 | (0.942-1.948) | 0.101 |
| Re-experiencing symptoms | 2.983 | (1.336-6.659) | 0.008 |

Note: aHR: Adjusted Hazard Ratio; 95% C.I.: 95% confidence interval; PTSD: posttraumatic stress disorder

*PTSD symptoms as moderated by genetic vulnerability to CI*: Since PTSD may also act as a vulnerability factor, we examined whether PTSD symptoms were moderated by APOE-ε4 status. In this case, table A3 revealed that no such association existed and that the trend was inverted in direction.

**Table A3.** Adjusted associations between posttraumatic stress disorder re-experiencing symptoms and its interaction with apolipoprotine-ε4 allele possession with incidence of cognitive impairment

| **Characteristic** | **aHR** | **95% C.I.** | **P** |
| --- | --- | --- | --- |
| Re-experiencing symptom severity | 3.480 | (1.608-7.533) | 0.002 |
| APOE-ε4 allele *vs.* none | 1.526 | (1.036-2.246) | 0.032 |
| APOE-ε4 allele x Re-experiencing symptom severity | 0.372 | (0.066-2.116) | 0.265 |

Note: aHR: Adjusted Hazard Ratio; PTSD: posttraumatic stress disorder; APOE-ε4: apolipoprotein-ε4 allele

*Biases in using in-clinic diagnosis:* CI was identified in the clinic at the time of visit, resulting in a potential for timing of measure to be associated with risk of CI thereby causing a biased estimate of the incidence rate. If this were a source of bias, then we would expect that scores at baseline or follow-up would be associated with cognitive score and/or for effects found using methods that ignore time to find different results. Examining the association between baseline MoCA score and timing revealed that there was no association between MoCA score and length of time until follow-up assessment (B = -0.000, *P*=0.213) nor between MoCA score at follow-up and length of time since baseline assessment (B = -0.000, *P* = 0.359). Additionally, replications of the above analysis examining predictors of the risk of having CI at follow-up using log-binomial models ^19^ revealed similar overall results to those presented in analyses of incidence shown in this study (Table A4).

**Table A4.** Adjusted associations between posttraumatic stress disorder re-experiencing symptoms and its interaction with apolipoprotine-ε4 allele possession with incidence of cognitive impairment

| **Characteristic** | **RR** | **95% C.I.** | **P** |
| --- | --- | --- | --- |
| Age, years | 1.014 | 0.996-1.032 | 0.119 |
| PTSD symptom severity | 2.151 | 1.178-3.928 | 0.013 |
| WTC exposure severity | 0.884 | 0.556-1.403 | 0.600 |
| WTC exposure severity x APOE-ε4 status | 3.719 | 1.876-7.371 | <0.001 |
| Any APOE-ε4 *vs.* no APOE-ε4 | 0.985 | 0.714-1.361 | 0.929 |
| Female *vs.* Male | 1.108 | 0.745-1.649 | 0.613 |
| Race/Ethnicity |  |  |  |
| White | 1.000 |  |  |
| Black | 1.947 | 1.255-3.019 | 0.003 |
| Other | 1.274 | 0.925-1.755 | 0.138 |
| Hispanic | 1.521 | 1.007-2.298 | 0.046 |
| Non-Traditional Responder vs. Law Enforcement | 1.203 | 0.915-1.581 | 0.186 |
| Educational Attainment |  |  |  |
| High School or Less | 1.000 |  |  |
| Some College | 0.967 | 0.73-1.281 | 0.816 |
| University Degree | 0.631 | 0.441-0.904 | 0.012 |
| Hypertension | 1.161 | 0.888-1.517 | 0.275 |
| Diabetes | 0.736 | 0.476-1.138 | 0.168 |
| Cardiovascular disease | 1.292 | 0.757-2.203 | 0.348 |
| All cause cancer | 0.940 | 0.676-1.307 | 0.714 |
| Head Injury |  |  |  |
| None | 1.000 |  |  |
| Non-concussive | 1.109 | 0.72-1.708 | 0.637 |
| Concussion | 0.654 | 0.328-1.305 | 0.229 |
| Loss of consciouness | 1.003 | 0.665-1.513 | 0.989 |
| Multiple | 1.138 | 0.809-1.601 | 0.456 |

**Note**: These results represent data from a log-binomial model. RR: Risk Ratio at follow-up; 95% C.I.: 95% confidence interval; PTSD: posttraumatic stress disorder; WTC: World Trade Center; APOE-ε4: apolipoprotein-ε4 allele

**References for Appendix**

1. Anstey KJ, Cherbuin N, Eramudugolla R, et al. Characterizing mild cognitive disorders in the young-old over 8 years: prevalence, estimated incidence, stability of diagnosis, and impact on IADLs. *Alzheimer's & Dementia.* 2013;9(6):640-648.

2. Bae JB, Kim YJ, Han JW, et al. Incidence of and risk factors for Alzheimer's disease and mild cognitive impairment in Korean elderly. *Dementia and geriatric cognitive disorders.* 2015;39(1-2):105-115.

3. Brodaty H, Heffernan M, Kochan NA, et al. Mild cognitive impairment in a community sample: the Sydney Memory and Ageing Study. *Alzheimer's & dementia.* 2013;9(3):310-317. e311.

4. Busse A, Bischkopf J, Riedel-Heller SG, Angermeyer MC. Mild cognitive impairment: prevalence and incidence according to different diagnostic criteria: Results of the Leipzig Longitudinal Study of the Aged (LEILA75+). *The British Journal of Psychiatry.* 2003;182(5):449-454.

5. Caracciolo B, Palmer K, Monastero R, Winblad B, Bäckman L, Fratiglioni L. Occurrence of cognitive impairment and dementia in the community: a 9-year-long prospective study. *Neurology.* 2008;70(19 Part 2):1778-1785.

6. Ganguli M, Fu B, Snitz BE, Hughes TF, Chang C-CH. Mild cognitive impairment: incidence and vascular risk factors in a population-based cohort. *Neurology.* 2013;80(23):2112-2120.

7. Katz MJ, Lipton RB, Hall CB, et al. Age and sex specific prevalence and incidence of mild cognitive impairment, dementia and Alzheimer’s dementia in blacks and whites: A report from the Einstein Aging Study. *Alzheimer disease and associated disorders.* 2012;26(4):335.

8. Manly JJ, Tang MX, Schupf N, Stern Y, Vonsattel JPG, Mayeux R. Frequency and course of mild cognitive impairment in a multiethnic community. *Annals of Neurology: Official Journal of the American Neurological Association and the Child Neurology Society.* 2008;63(4):494-506.

9. Ravaglia G, Forti P, Montesi F, et al. Mild cognitive impairment: epidemiology and dementia risk in an elderly Italian population. *J Am Geriatr Soc.* 2008;56(1):51-58.

10. Roberts R, Knopman DS. Classification and epidemiology of MCI. *Clin Geriatr Med.* 2013;29(4):753-772.

11. Solfrizzi V, Panza F, Colacicco A, et al. Vascular risk factors, incidence of MCI, and rates of progression to dementia. *Neurology.* 2004;63(10):1882-1891.

12. Tervo S, Kivipelto M, Hänninen T, et al. Incidence and risk factors for mild cognitive impairment: a population-based three-year follow-up study of cognitively healthy elderly subjects. *Dementia and geriatric cognitive disorders.* 2004;17(3):196-203.

13. Unverzagt FW, Ogunniyi A, Taler V, et al. Incidence and risk factors for cognitive impairment no dementia and mild cognitive impairment in African Americans. *Alzheimer disease and associated disorders.* 2011;25(1):4.

14. Au B, Dale-McGrath S, Tierney MC. Sex differences in the prevalence and incidence of mild cognitive impairment: a meta-analysis. *Ageing research reviews.* 2017;35:176-199.

15. Ward A, Arrighi HM, Michels S, Cedarbaum JM. Mild cognitive impairment: disparity of incidence and prevalence estimates. *Alzheimers Dement.* 2012;8(1):14-21.

16. Miettinen O. Estimability and estimation in case-referent studies. *American journal of epidemiology.* 1976;103(2):226-235.

17. Hedges LV, Vevea JL. Fixed- and random-effects models in meta-analysis. *Psychological Methods.* 1998;3(4):486-504.

18. Luft BJ, Schechter C, Kotov R, et al. Exposure, probable PTSD and lower respiratory illness among World Trade Center rescue, recovery and clean-up workers. *Psychol Med.* 2012;42(5):1069-1079.

19. McNutt LA, Wu C, Xue X, Hafner JP. Estimating the relative risk in cohort studies and clinical trials of common outcomes. *Am J Epidemiol.* 2003;157(10):940-943.
