# Supplementary figures and images for: Incidence of mild cognitive impairment in World Trade Center responders: Long-term consequences of re-experiencing the events on 9/11/2001
Source: Alzheimers Dement (Amst). 2019 Sep 6;11:628–36. doi: 10.1016/j.dadm.2019.07.006 (PMC6733774; doi:10.1016/j.dadm.2019.07.006)

**Supplemental Figure 1. Sampling method and criteria**

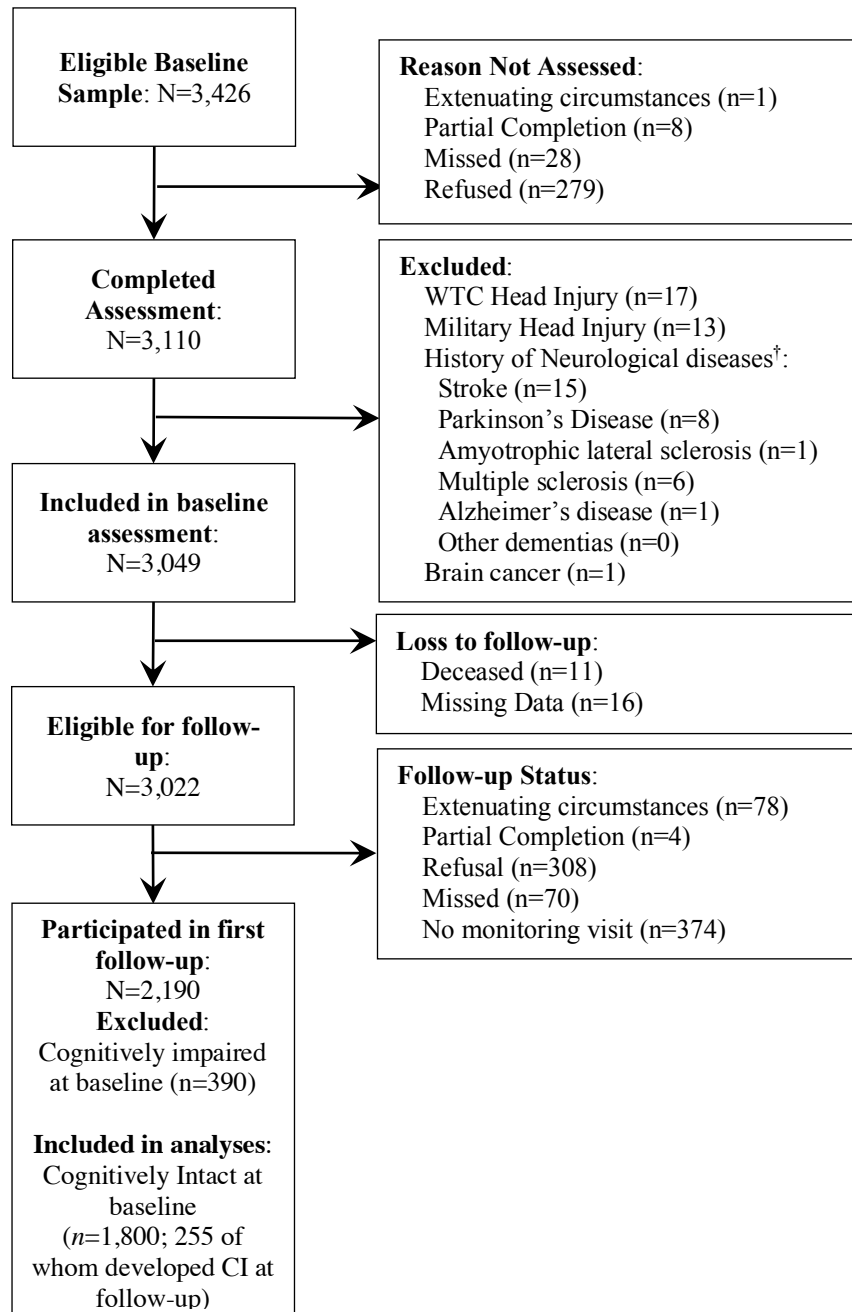

Supplement: Supplementary Fig. 1 [file mmc2.pdf]
